# Supplementary material for: Atomic stiffness for bulk modulus prediction and high-throughput screening of ultraincompressible crystals
Source: Nat Commun. 2023 Jul 17;14:4258. doi: 10.1038/s41467-023-39826-2 (PMC10352355; doi:10.1038/s41467-023-39826-2)
Supplement: Supplementary file 1 — Supplementary Information [file 41467_2023_39826_MOESM1_ESM.pdf]

Supplementary Information for

Atomic stiffness for bulk modulus prediction and high-throughput screening of ultraincompressible crystals

Ruihua Jin[1#], Xiaoang Yuan[1#], and Enlai Gao[1*]

[1]Department of Engineering Mechanics, Wuhan University, Wuhan, Hubei 430072, China.

[#]R.J. and X.Y. contributed equally.

[*]Corresponding author. Email: enlaigao@whu.edu.cn

This PDF file includes:

Supplementary Figures 1 to 3.
Supplementary Tables 1 to 2.
Supplementary References

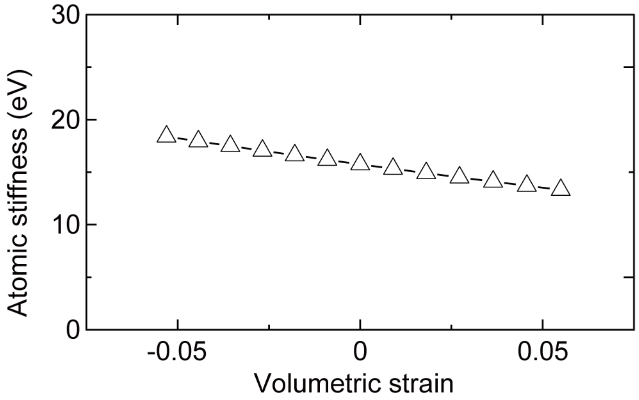

Supplementary Figure 1. Atomic stiffness of diamond as a function of volumetric strain. The atomic stiffness is calculated from second derivative of atomic energy with respect to volumetric strain. Source data are provided as a Source Data file.

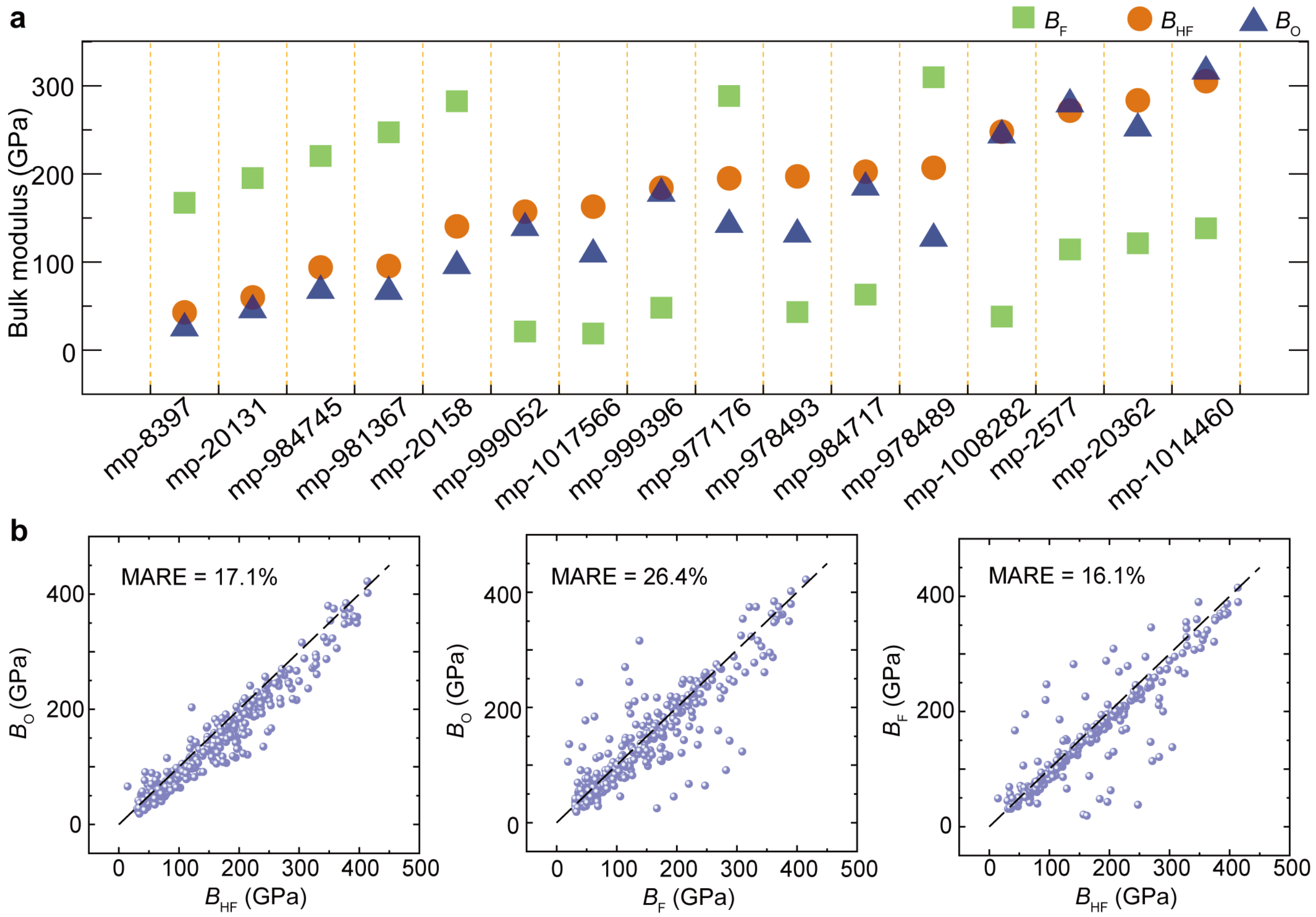

Supplementary Figure 2. Comparison of bulk moduli from our formula prediction (B_O), first-principles calculations in MP database (B_F), and high-fidelity first-principles calculations (B_{HF}). a, Bulk moduli from our formula prediction (B_O), first-principles calculations in MP database (B_F), and high-fidelity first-principles calculations (B_{HF}) for 16 crystals having a large difference between B_O and B_F (> 80 GPa), **b,** and for 306 crystals randomly selected from 6192 crystals. Source data are provided as a Source Data file.

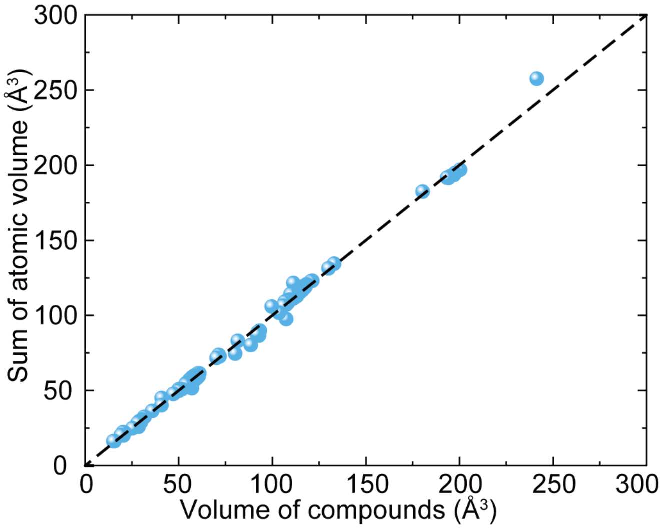

Supplementary Figure 3. Comparison of the sum of atomic volume and the actual total volume of compounds with predicted bulk moduli from our formula (B_O) higher than 350 GPa in Materials Project (MP) and Open Quantum Materials Database (OQMD). Source data are provided as a Source Data file.

Supplementary Table 1. Comparison of bulk moduli predicted from our formula (B_O), first-principles calculations in MP database (B_F), and high-fidelity first-principles calculations (B_{HF}) for 16 crystals having a large difference of bulk moduli between our formula prediction and first-principles calculations in MP database.

MP-ID	Formula	B_F (GPa)	B_O (GPa)	B_{HF} (GPa)
MP-8397	$CsSrF_3$	167	25	43
MP-20131	YIn_3	195	45	60
MP-984745	$CsSnO_3$	220	67	94
MP-981367	$SrTlO_3$	247	66	95
MP-20158	PbO_2	282	95	140
MP-999052	Ti_2MnAl	21	139	157
MP-1017566	$GePbO_3$	19	108	162
MP-999396	Nb_3Ni	48	177	184
MP-977176	$NaTcO_3$	288	142	195
MP-978493	$SiSnO_3$	43	131	197
MP-984717	$BeNi_3$	63	184	202
MP-978489	$SiPbO_3$	309	126	207
MP-1008282	Cr_3Fe	38	244	248
MP-2577	Mn_3Rh	114	278	271
MP-20362	Mn_3NiN	121	251	283
MP-1014460	Cr_3N_4	138	316	305

Supplementary Table 2. Comparison of bulk moduli from our formula (B_O) and experimental measurements (B_{EXP}) [1-17].

Crystal	Atomic volume (Å^3)	B_O (GPa)	B_{EXP} (GPa)
Diamond	5.71	436	442 [1]
BN	5.96	348	369 [2]
OsN_2	9.11	402	358 [3]
PtN_2	9.24	329	354 [4]
MoN	10.14	334	345 [5]
TaC	11.05	273	344 [6]
WC	10.38	357	329 [6]
Mo_2N	12.13	301	301 [5]
NbC	11.16	354	300 [6]
RuO_2	10.45	252	270 [7]
HfC	12.48	197	242 [6]
TiC	10.16	219	242 [8]
β-SiN_4	10.39	214	234 [9]
$ZrSiO_4$	9.72	183	230 [10]
Cr_2O_3	9.64	202	224 [11]
ZrC	13.10	179	223 [8]
SiC	10.50	199	211 [12]
AlN	10.63	187	201 [13]
GaN	11.74	154	180 [13]
BP	11.75	136	173 [14]
MgO	9.64	106	156 [15]
InN	16.13	114	139 [13]
CaO	14.17	68	106 [11]
BeS	14.48	61	105 [16]
Si	20.44	83	100 [17]

Supplementary References

1 McSkimin, H. J. & Andreatch, P. Elastic moduli of diamond as a function of pressure and temperature. *J. Appl. Phys.* **43**, 2944-2948 (1972).

2 Knittle, E., Wentzcovitch, R. M., Jeanloz, R. & Cohen, M. L. Experimental and theoretical equation of state of cubic boron nitride. *Nature* **337**, 349-352 (1989).

3 Young, A. F. *et al.* Synthesis of novel transition metal nitrides IrN_2 and OsN_2. *Phys Rev Lett*. **96**, 155501 (2006).

4 Gregoryanz, E. *et al.* Synthesis and characterization of a binary noble metal nitride. *Nat. Mater*. **3**, 294-297 (2004).

5 Soignard, E. *et al.* High-pressure synthesis and study of low-compressibility molybdenum nitride (MoN and MoN_{1-x}) phases. *Phys. Rev. B* **68**, 132101 (2003).

6 Brown, H. L., Armstrong, P. E. & Kempter, C. P. Elastic properties of some polycrystalline transition-Metal monocarbides. *J. Chem. Phys*. **45**, 547-549 (1966).

7 Hazen, R. M. & Finger, L. W. Bulk moduli and high-pressure crystal structures of rutile-type compounds. *J. Phys. Chem. Solids* **42**, 143-151 (1981).

8 Chang, R. & Graham, L. J. Low-temperature elastic properties of ZrC and TiC. *J. Appl. Phys.* **37**, 3778-3783 (1966).

9 Zerr, A. *et al.* Elastic moduli and hardness of cubic silicon nitride. *J. Am. Ceram. Soc.* **85**, 86-90 (2004).

10 Errandonea, D. & Manjón, F. J. Pressure effects on the structural and electronic properties of ABX_4 scintillating crystals. *Prog. Mater. Sci.* **53**, 711-773 (2008).

11 Anderson, D. L. & Anderson, O. L. Brief report: The bulk modulus-volume relationship for oxides. *J. Geophys. Res*. **75**, 3494-3500 (1970).

12 Lam, P. K., Cohen, M. L. & Martinez, G. Analytic relation between bulk moduli and lattice constants. *Phys. Rev. B* **35**, 9190-9194 (1987).

13 Wright, A. F. Elastic properties of zinc-blende and wurtzite AlN, GaN, and InN. *J. Appl. Phys.* **82**, 2833-2839 (1997).

14 Wettling, W. & Windscheif, J. Elastic constants and refractive index of boron phosphide. *Solid State Commun.* **50**, 33-34 (1984).

15 Mao, H. K. & Bell, P. M. Equations of state of MgO and ε Fe under static pressure conditions. *J. Geophys. Res*. **84**, 4533 (1979).

16 Khenata, R. *et al.* Elastic and optical properties of BeS, BeSe and BeTe under pressure. *Solid State Electron.* **50**, 1382-1388 (2006).

17 Caus, M., Dovesi, R. & Roetti, C. Pseudopotential Hartree-Fock study of seventeen III-V and IV-IV semiconductors. *Phys. Rev. B* **43**, 11937-11943 (1991).
