# Supplementary material for: Atomic stiffness for bulk modulus prediction and high-throughput screening of ultraincompressible crystals
Source: Nat Commun. 2023 Jul 17;14:4258. doi: 10.1038/s41467-023-39826-2 (PMC10352355; doi:10.1038/s41467-023-39826-2)
Supplement: Supplementary file 3 — Description of Additional Supplementary Files [file 41467_2023_39826_MOESM3_ESM.pdf]

Description of Additional Supplementary Files

File Name: Supplementary Data 1

Description: Atomic stiffnesses of different allotropes for each element within the periodic table. The mean absolute relative error (MARE) is calculated with respect to the corresponding dense crystalline form.

File Name: Supplementary Data 2

Description: Comparison of the predicted bulk moduli for the top 50 compounds with the largest bulk modulus listed in the relevant literature.

File Name: Supplementary Data 3

Description: Atomic coordinates of the optimized structures for the discovered ultra-incompressible crystals.
